# Key Insights From the International ICU Diary Conference 2025

**DOI:** 10.1111/nicc.70430

**Published:** 2026-03-30

**Authors:** Peter Nydahl, Bev Ewens, Anne‐Sophie Debue, Xavier Fiorilla, Kristin Gabriel, Alessandro Galazzi, Louise Gallie, Nikolas Groth, Carly Hickey, Mark Hudson, Fiona Lynch, Nicola Maxwell, Margo van Mol, Caroline Renner, Louise Rose, Kate Tantam, Lioudmila Karnatovskaia, Christina Jones

**Affiliations:** ^1^ Nursing Research University Hospital of Schleswig‐Holstein Kiel Germany; ^2^ Institute of Nursing Science and Practice Paracelsus Medical University Salzburg Austria; ^3^ School of Nursing and Midwifery Edith Cowan University Perth Western Australia Australia; ^4^ Better Support Department, 101 fund Paris France; ^5^ Sir Charles Gairdner Hospital Perth Western Australia Australia; ^6^ Family Representative Berlin Germany; ^7^ Department of Medicine and Surgery LUM University Casamassima Italy; ^8^ Patient Representative, Torpoint Cornwall UK; ^9^ Department of Medicine MSB Medical School Berlin Berlin Germany; ^10^ University of Toronto Toronto Ontario Canada; ^11^ Patient Representative Glasgow UK; ^12^ Great Ormond Street Hospital London UK; ^13^ Intensive Care Adults, Erasmus Medical Centre Rotterdam the Netherlands; ^14^ Department of Neurology and Neuro‐Rehabilitation Herz‐Kreislauf‐Zentrum, Klinikum Hersfeld Rotenburg GmbH Rotenburg a. F Germany; ^15^ Justus Liebig University Giessen Germany; ^16^ Florence Nightingale Faculty of Nursing, Midwifery and Palliative Care, King's College London London UK; ^17^ Rehabilitation Team, Critical Care University Hospitals Plymouth NHS Trust Plymouth UK; ^18^ Pulmonary & Critical Care Medicine, Mayo Clinic Rochester Minnesota USA; ^19^ ICUsteps Support Charity London UK

**Keywords:** diary, family, humanising, intensive care unit, patient‐centredness

## Abstract

We report on the 2025 International ICU Diary Conference that provided a comprehensive update on current knowledge, innovations and global practices related to ICU diaries. Held in a 6‐week online format, the event featured 23 speakers and was attended by mean 69.7 (SD 20.5) participants per session. Topics included development of ICU diaries, use in special settings, digital diaries, survivors' and families' experiences, Post‐Intensive‐Care Syndrome and follow‐up and implementation projects. Highlights were innovative solutions for digital diaries and the report of persons with lived and living experiences with diaries, changing the focus on humanising critical care.

**Relevance for Practice:**

ICU diaries support patient‐ and family‐centred care and should be implemented more widely across healthcare systems. Their successful adoption requires contextual adaptation and policy support, with future development likely to be shaped by the increasing use of digital ICU diaries.

## Impact Statements

**What is known about this topic?:**

- ICU diaries are recommended in several professional society guidelines to improve psychological outcomes and support recovery after critical illness.
- They contribute to patient‐ and family‐centred care, particularly in addressing Post‐Intensive‐Care Syndrome.
- Implementation remains inconsistent and is currently limited to specific regions and healthcare settings.

**What this paper adds:**

- Provides a comprehensive international overview of current practices, innovations, and challenges in ICU diary use based on a global online conference.
- Highlights the growing role of digital ICU diaries and the need for adaptation to diverse patient groups and care contexts.
- Emphasizes the importance of lived experiences and international collaboration in advancing and humanising critical care practices.

## 1 Background

Intensive care unit (ICU) diaries are written in plain language for and to critically ill patients during their ICU stay [1]. These diaries, typically authored by healthcare professionals and family members, document the patient's journey of critical illness from admission to discharge. Current knowledge suggests that recall of nightmare‐like delusional memories of ICU can potentiate psychological complications. Therefore, ICU diaries aim to reduce this potential psychological sequelae by helping patients and their families reconstruct a coherent memory of their ICU stay [2]. Diary content includes an explanation of reasons for hospital and ICU admission, daily entries describing the patient's current status, clinical developments and observations. Optional additions include photographs, a get‐to‐know‐me page, a glossary explaining medical terms, personal biographies, contact details and other topics [3]. Diaries may be paper written or in digital format. They are usually provided back to patients after the acute phase, once they are recovering [4]. Diary handover requires sensitive timing and support, as reading them can evoke intense emotions in patients and in family members. Although some patients do not wish to read their diary, most perceive it as helpful in understanding their experience [5]. Family members also value the diary as a means of communication and emotional processing.

Recent guidelines for adult Post‐Intensive‐Care Syndrome (PICS) and family integration suggest that use of ICU diaries can reduce post‐traumatic stress disorder (PTSD) and depression in survivors of critical illness [6, 7]. Qualitative studies recruiting patients report improved memory reconstruction, emotional processing and connection with healthcare providers [8]. However, for families, the impact is more nuanced with no consistent reduction in psychological distress from quantitative data, but qualitative studies highlighting improved coping, stronger communication and potential for post‐traumatic growth [9]. Additionally, healthcare professionals describe diaries as tools that enhance reflective practice, foster empathy and humanise ICU treatment and care, though they may also pose an emotional and cognitive burden [9]. Especially during the Covid‐19 pandemic, digital diaries were increasingly developed and used, but they also raised questions for data protection, access regulation, privacy and others [4].

## 2 Aims

This conference had multiple aims: (a) providing an overview of the current knowledge, recent innovations and current clinical practice regarding ICU diaries, (b) discussing implementation in diverse settings, and (c) to stimulate international collaboration and discussion.

## 3 Methods

Based on positive experiences of former diary conferences, five experts—three nurses, one intensivist and one former ICU patient representing three continents—planned and organised the conference [1, 10]. Conference structure and content were defined over the course of three planning meetings. The conference was free of charge for participants and hosted via Zoom Pro (Zoom Communications, San Jose, USA). The structure included a weekly series of 90‐min online sessions over five consecutive weeks, with a sixth session added based on demand of participants.

Speakers were selected by the organising team based on recent publications, expert recommendations and current social media discussions relating to ICU diaries. All agreed to participate without financial compensation. Each 90‐min session included: a brief introduction by the chairs, short presentations (approx. 10 min each) totalling 50–60 min, 30‐min audience discussion and closing wrap‐up with information on the next session. The sessions were narratively summarised by the organisational team and reviewed by the speakers; due to the limited space of this report, only selected references could be cited. The conference publicity was disseminated via the international ICU Diary Network, social media and network's newsletters. Programme details and registration were available at www.icu‐diary.org.

Sessions were evaluated via anonymous online surveys (SurveyMonkey) using scales from 0 to 10 (with 10 representing best satisfaction with experiences) and a comment field. The evaluation form contained six questions: (a) overall experience, (b) sharing knowledge and experiences, (c) raising awareness for PICS and diaries, (d) supporting clinicians in implementing diaries, (e) highlight stories and experiences from patients, families and clinicians, and (f) technical experience, and an option for comments. Evaluation survey links were shared during each session.

## 4 Results

The international ICU Diary conference was held over June and July 2025. In total, six sessions were held (Table 1), with 23 speakers sharing their knowledge, expertise and experiences with ICU diaries. In total, *n* = 418 participants joined all sessions, with a mean of 69.7 (SD 20.5) participants in each session. Comments from participants are reported in Table S1. The topic and content of each session were as follows:

**TABLE 1:** Conference content.

Sessions	Titles	Contents
1	ICU diaries	Development and photos of ICU diaries, Evidence of diaries, Diaries in the United States of America, Diaries in Australia, Diary example, Patient's perspective
2	Diary implementation in special settings	Palliative care, Paediatric ICU, Delirium experiences, Australian perspective, Restarting ICU diaries after COVID
3	Digital diaries	Digital diaries in the Netherlands, Digital diaries in France, Digital diaries in United Kingdom, Digital diaries in Germany, Digital diaries in Italy
4	Lived experiences of diaries	Personal experiences by three former patients with critical care experience, and one former bereaved relative
5	PICS and after care/follow‐up	Introduction into post intensive care syndrome, Follow‐up in United Kingdom, Follow‐up in the United States of America, Follow‐up in Australia
6	How to write and implement ICU diaries and humanised critical care	How to write diaries, How to implement diaries, How to implement humanised critical care, How to evaluate implementation

### 4.1 Session 1: ICU Diaries—An Introduction

Presentations highlighted the evolution and international adoption of ICU diaries, including recent developments such as photo diaries, digital formats and structured journals with educational content. Current knowledge was presented that demonstrates diaries reduce PTSD and depression in patients and supports emotional coping for families and healthcare professionals, though effects on family distress are inconsistent [11]. Usage remains variable across countries, hindered by limited awareness, staffing constraints and lack of standardised adoption protocols. Recent innovations include AI‐generated subtitles for virtual diary conferences, integrated handbook sections about diaries and print‐on‐demand diaries [3]. These developments reflect a growing interest in humanising intensive care through accessible, reflective, cultural and context specific adaptable diary formats [12].

### 4.2 Session 2: Diary Implementation in Special Settings

Presentations explored innovative and underrepresented uses of ICU diaries including palliative care to support bereaved families and staff, paediatric ICU, patients with delirium, as well as barriers to diary use [13, 14, 15]. COVID‐19 led to a shift towards digital diaries, though many ICUs have reintroduced structured paper formats with infection‐safe designs [4]. Diaries are increasingly viewed as tools for both patient recovery and staff reflection. New developments include a national implementation guideline in Norway, standardised notebook formats and expansion of ICU diary communities of practice with the aim of fostering consistency, accessibility and sustainable usage.

### 4.3 Session 3: Digital Diaries

Presentations explored recent developments across Europe highlighting various models of digital ICU diaries that emphasise accessibility, data security and personalisation [4]. In the Netherlands and France, diaries are integrated via web apps (e.g., Post‐IC and LifeMapp Diary). These offer user‐friendly, General Data Protection Regulation (GDPR) compliant platforms with multimedia support. In the United Kingdom, robust implementation strategies, co‐design and use of a GDPR compliant platform boosted engagement. In Germany, a start‐up company enables automated diary generation, private entries accessible only to patients and printable archives via a tablet; data are stored on a central server in Germany, encrypted and GDPR‐compliant. Common barriers include technology access, staff hesitation and administrative burden. However, tailored training, prompting strategies and integration into existing workflows can enhance acceptance and sustainability [4, 16, 17]. Digital diaries offer great opportunities to strengthen ICU‐diary‐focused research by allowing some standardisation to the intervention and a facilitated evaluation of quantitative indicators.

### 4.4 Session 4: Lived Experiences of Diaries

Presentations included personal accounts revealing the powerful yet varied effects that ICU diaries have on survivors and their family members. One patient described struggling with unanswered questions and post‐ICU trauma when only an official, impersonal diary was available, while their relative‐created diary offered comfort [18]. Another relative found writing deeply grounding during her partner's coma and death, transforming helplessness into meaningful connection. These testimonies highlighted diaries as emotional anchors, bridging fragmented memory, fostering communication and supporting long‐term psychological healing in profoundly personal ways. Another former patient highlighted the lasting psychological burden after ICU, including PTSD triggered by untreated delirium and the lack of structured follow‐up. Diaries, she argued, could offer continuity and explanation during recovery [19]. Collectively, these stories revealed how personalised diaries supported identity, emotional healing and meaning making.

### 4.5 Session 5: PICS and After Care/Follow‐Up

Presentations highlighted innovations in ICU follow‐up and rehabilitation, emphasising the integration of diaries into structured post‐ICU care [6]. New approaches include co‐designed diary packs with photo prompts, digital education tools and systematic screening for PICS across care phases. In the United Kingdom, there are national standards and a coordinated rehabilitation framework for ICU recovery resulting in recovery services being provided by ~70% of ICU services across the country [20]. In contrast, Australian recovery clinics explore risk‐targeted models and peer support groups [11]. Despite persistent gaps in access and funding, recent efforts aim to personalise care, reduce fragmentation and embed diaries into multidisciplinary pathways to enhance psychological recovery and continuity of care after ICU [21].

### 4.6 Session 6: How to Write and Implement ICU Diaries and Humanised Critical Care

Presentations addressed practical writing guidance, implementation strategies and ICU diary evaluation. New tools (e.g., digital diaries and/or automated correction) support writing for varied literacy levels, use respectful language around death and include meaningful photographs with retrospective consent. A global overview of diary implementation reports varied uptake, with solutions including policy templates, education and family involvement. Novel innovations like the ‘Get to Know Me Board’ humanise care and promote dignity, especially in delirium prevention and goal‐concordant decision‐making [12]. Several strategies can be used for evaluation, such as incorporating narrative analysis, audit cycles and international benchmarks [22]. Diaries are increasingly recognised as acts of care quality fostering empathy, ethical awareness and staff satisfaction.

## 5 Evaluation

**FIGURE 1:**
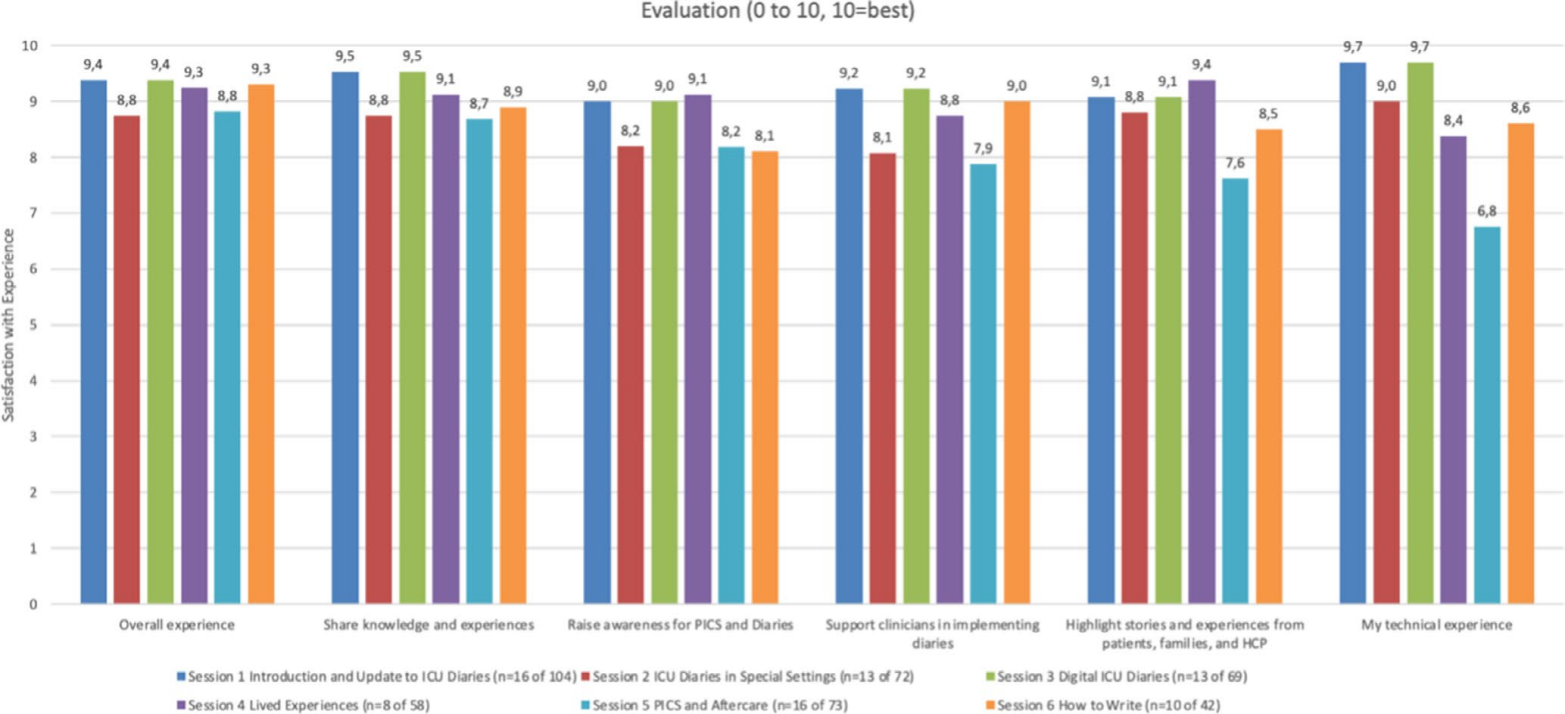
Conference evaluation.

A total of 18.2% (76/418) of participants took part in six evaluations. The overall conference experience was rated as mean 9.1 (SD 0.3) out of 10 (Figure 1). The evaluations of the individual days showed variations, reflecting the differing topics covered. Section 3 ‘digital ICU diaries’ received the highest satisfaction with experience of 9.3 (SD 0.3).

## 6 Conclusions

The 2025 International ICU Diary Conference successfully brought together clinicians, researchers, former patients and family members from around the world in a new, inclusive online format. Spanning 6 weeks, the conference highlighted major developments in ICU diary practice from digital innovations and structured formats to integration into follow‐up care and national guidelines. Personal testimonies underscored the value of diaries in psychological recovery, identity reconstruction and family support. Especially the topics digital diaries, experiences of patients and families, and implementation into practice across different settings were intensely discussed and should be further elaborated in future conferences. Evaluation of the conference was positive, with high engagement and content relevance. The virtual format had the benefit of enabling participants to connect with each other through the chat function, which could lead to future international collaborations. The current knowledge and global interest confirm the need for wider implementation and consistent use of ICU diaries in critical care settings. Continued international collaboration is essential to learn from each other and to close remaining gaps in practice. Planning is already underway for the next virtual ICU Diary Conference within the next 1 or 2 years.

## Funding

The authors have nothing to report.

## Ethics Statement

The authors have nothing to report.

## Consent

Former patients and one family member are involved as co‐authors in this work and gave consent to publication.

## Conflicts of Interest

Peter Nydahl, Bev Ewens, Xavier Fiorilla, Kristin Gabriel, Alessandro Galazzi, Louise Gallie, Mark Hudson, Nicola Maxwell, Margo van Mol, Lioudmila Karnatovskaia, Christina Jones and Caroline Renner report no conflicts. Anne‐Sophie Debue is a full‐time employee of the 101 Fund, the non‐profit organisation that has developed the free ICU diary web‐application LifeMapp Diary. Nikolas Groth is the founder of IntensivKontakt, a provider of digital ICU communication systems. Louise Rose is a co‐founder of Life Lines that received philanthropic contributions to support virtual visiting and the bespoke ICU e‐diary described in this publication. Carly Hickey has a consulting company, distributing diaries.

## Supporting information


**Table S1:** Comments by participants.

## Data Availability

Data sharing not applicable to this article as no datasets were generated or analysed during the current study.
