# Supplementary material for: Key Insights From the International ICU Diary Conference 2025
Source: Nurs Crit Care. 2026 Mar 30;31(3):e70430. doi: 10.1111/nicc.70430 (PMC13035930; doi:10.1111/nicc.70430)
Supplement: Supplementary file 1 — Table S1: Comments by participants. [file NICC-31-0-s001.docx]

**SUPPLEMENT**

**Key Insights from the International ICU Diary Conference 2025**

Nydahl P, Ewens B, Debue A-S, Fiorilla X, Gabriel K, Galazzi A, Gallie L, Groth N, Hickey C, Hudson M, Lynch F, Maxwell N, van Mol M, Renner C, Rose L, Tantam K, Karnatovskaia L, Jones C.

Nursing in Critical Care, 2026

**Table S1**: **Comments by participants**

| June 10^th^ |
| --- |
| - I found the meeting very helpful and insightful. Thank you for organising it. |
| - Thank you. |
| June 17^th^ |
| - I have still more questions to ask about the storage, how long can we store, how to dispose if not going to hand over and teaching materials etc |
| - Very interesting to listen to different experiences. Many thanks to all |
| June 24^th^ |
| - Excellent conference in which I am able to share valuable information with my colleagues. Thank you |
| - I’m honestly so impressed and grateful to all of you who coordinate this digital conference, and for the high calibre of the presentations given by the speakers - thank you |
| - Very interesting, will be something I would hope to bring to my unit |
| July 1^st^ |
| - Thank you - what a great insightful session |
| - The best one so far thank you |
| July 8^th^ |
| - The tech delays cannot be helped, but they did impact on the experience. Having said that, it was an amazing opportunity to hear from International experts from the comfort of my home. Thank you for organising such a great series. |
| - It's very beautiful to see many professionist from all the word talk about the future ad the implementation of this instrument, I’m an Italian nursing student and I'm doing my graduation thesis about ICU diary, I really appreciate this conference. Thanks! |
| - it was great thank you but could not hear the patient film which was a shame |
| - Thank you for sharing the knowledge about the patient diaries across from the globe. Excellent work. Thank you |
| - One of the sessions the sound kept coming in and out - made it very hard to follow |
| July 15^th^ |
| - Due to signal dropping out I missed out on some of the conversations in the chat, as well as links and certificate (as these are not available when logging back in). Could these be made available separately? Really appreciate all the work involved and this continues to inspire me on ending diaries are available to patient and families! Well done to all. |
| - A different time zone please! (I am in Australia!) |
| - Wonderful idea. If the conference repeats, I will join every session. |
| - Thank you so very much. I unfortunately did not find about the conference in time to attend any of the earlier sessions. This final session was excellent, inspiring, practical, evidence-based but also translational. Thank you! |
| - Thank You |
